# Supplementary material for: Balance between Pro- and Antifibrotic Proteins in Mesenchymal Stromal Cell Secretome Fractions Revealed by Proteome and Cell Subpopulation Analysis
Source: Int J Mol Sci. 2023 Dec 25;25(1):290. doi: 10.3390/ijms25010290 (PMC10779358; doi:10.3390/ijms25010290)
Supplement: Supplementary file 1 [file ijms-25-00290-s001.zip › ijms-2714092-supplementary.pdf]

Suplementary material

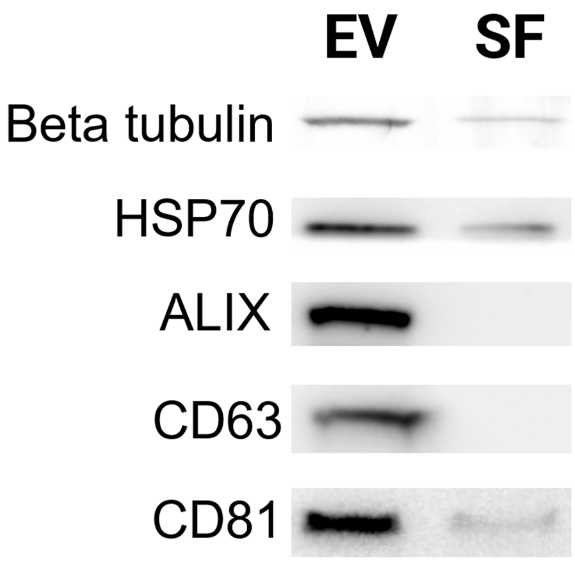

Figure S1. Extracellular vesicle proteins content differs between EV and SF subfractions. Western blotting.

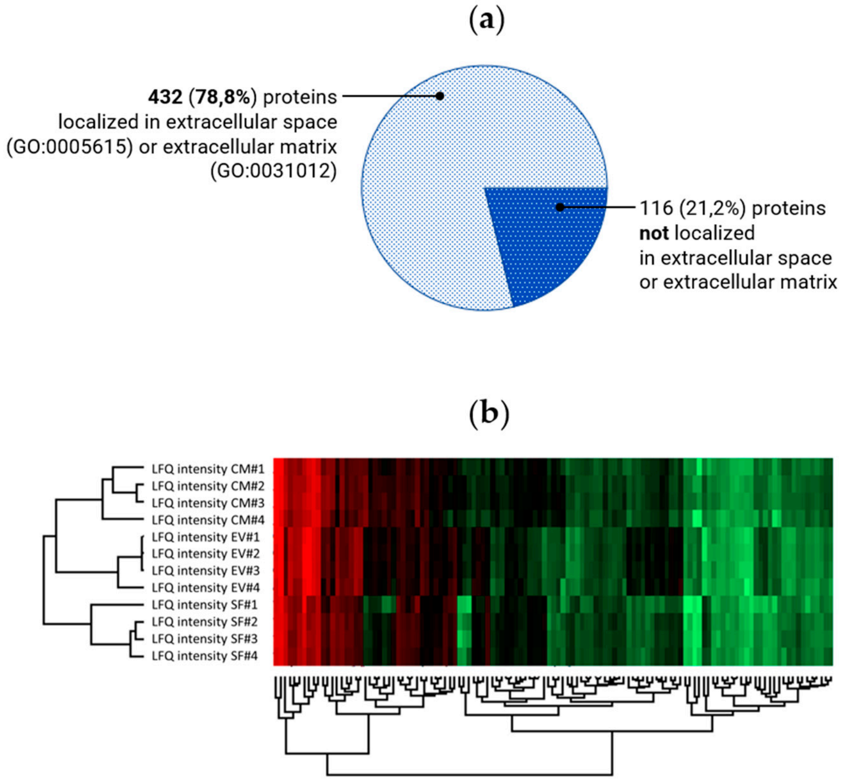

Figure S2. (**a**) MSC secretome fractions are enriched in secreted proteins. Round diagram representing content of proteins annotated as localized in the extracellular space (GeneOnthology term: 0005615, extracellular space) or in the extracellular matrix (GeneOnthology term: 0031012, extracellular matrix). (**b**) Hierarchical clustering of proteomic analysis results demonstrated that samples from the same MSC secretome fraction are clustered together.

Table S1. Enrichment of EV and SF subfractions compared to CM fraction in secreted proteins.

Protein name	Gene name	Enrichment relative to CM fraction, fold	
		for EV subfraction	for SF subfraction
1,4-alpha-glucan-branching enzyme	*GBE1*	not identified in CM	
14-3-3 protein beta/alpha	*YWHAB*	2.2	1.8
14-3-3 protein eta	*YWHAH*	4.0	2.1
40S ribosomal protein S16	*RPS16*	detected in EV only	
40S ribosomal protein S3	*RPS3*	4.1	2.3
40S ribosomal protein S8	*RPS8*	2.8	2.3
6-phosphogluconate dehydrogenase, decarboxylating	*PGD*	detected in SF only	
60 kDa heat shock protein, mitochondrial	*HSPD1*	detected in SF only	
60 kDa heat shock protein, mitochondrial	*HSPD1*	detected in SF only	
60S ribosomal protein L14	*RPL14*	detected in EV only	
60S ribosomal protein L5	*RPL5*	3.0	1.6
78 kDa glucose-regulated protein	*HSPA5*	2.5	1.1
Actin-related protein 2/3 complex subunit 4	*ARPC4*	1.2	2.5
Adenylyl cyclase-associated protein 1	*CAP1*	detected in SF only	
Adipocyte enhancer-binding protein 1	*AEBP1*	3.5	0.4
Agrin	*AGRN*	2.0	1.8
Alpha-actinin-1	*ACTN1*	not detected in CM	
Alpha-actinin-4	*ACTN4*	5.3	2.8
Angiopoietin-related protein 2	*ANGPTL2*	3.5	1.0
Angiopoietin-related protein 4	*ANGPTL4*	2.8	1.4
Annexin A1;Annexin	*ANXA1*	4.0	1.4
Annexin A2;Annexin	*ANXA2*	5.2	1.6
Annexin A5;Annexin	*ANXA5*	2.1	0.2
Brain acid soluble protein 1	*BASP1*	4.5	3.7
Calpain-2 catalytic subunit	*CAPN2*	detected in EV only	
Calreticulin	*CALR*	4.3	3.8
Carbohydrate sulfotransferase 14	*CHST14*	not detected in CM	
Coiled-coil domain-containing protein 80	*CCDC80*	3.2	1.2
Collagen alpha-1(I) chain	*COL1A1*	2.6	2.0
Collagen alpha-1(III) chain	*COL3A1*	2.7	2.3
Collagen alpha-1(XII) chain	*COL12A1*	2.1	1.2
Collagen alpha-2(IV) chain	*COL4A2*	3.9	1.3
Collagen alpha-2(V) chain	*COL5A2*	8.6	1.7
Collagen alpha-2(VI) chain	*COL6A2*	3.0	1.5
Collectin-12	*COLEC12*	0.2	2.4

Complement component 1 Q subcomponent-binding protein, mitochondrial	*C1QBP*	0.8	4.8
Cytoplasmic dynein 1 heavy chain 1	*DYNC1H1*	detected in SF only	
Cytoskeleton-associated protein 4	*CKAP4*	detected in EV only	
Dickkopf-related protein 3	*DKK3*	1.5	2.1
Dihydropyrimidinase-related protein 3	*DPYSL3*	2.2	1.3
Ectonucleotide pyrophosphatase/phosphodiesterase family member 2	*ENPP2*	0.7	2.2
EGF-containing fibulin-like extracellular matrix protein 2	*EFEMP2*	11.6	1.2
EMILIN-1	*EMILIN1*	2.6	0.4
Endoplasmin	*HSP90B1*	2.7	1.1
Endosialin	*CD248*	1.9	3.3
Eukaryotic translation initiation factor 2 subunit 3	*EIF2S3*	detected in SF only	
Extracellular serine/threonine protein kinase FAM20C	*FAM20C*	4.3	0.7
Extracellular sulfatase Sulf-1	*SULF1*	2.5	0.4
F-actin-capping protein subunit alpha-1	*CAPZA1*	detected in SF only	
Fibulin-1	*FBLN1*	3.3	0.1
Fibulin-5	*FBLN5*	3.9	1.1
Filamin-A	*FLNA*	5.0	2.1
Fructose-bisphosphate aldolase C	*ALDOC*	not detected in CM	
Glucosamine-6-phosphate isomerase 1	*GNPDA1*	detected in EV only	
Glucose-6-phosphate 1-dehydrogenase	*G6PD*	detected in EV only	
Glutathione S-transferase omega-1	*GSTO1*	5.4	1.1
Golgi apparatus protein 1	*GLG1*	detected in EV only	
Growth arrest-specific protein 6	*GAS6*	detected in SF only	
Growth arrest-specific protein 6	*GAS6*	detected in SF only	
Heat shock cognate 71 kDa protein	*HSPA8*	4.6	1.0
Heat shock protein HSP 90-beta	*HSP90AB1*	3.4	3.3
Hepatocyte growth factor	*HGF*	detected in EV only	
Heterogeneous nuclear ribonucleoprotein A1	*HNRNPA1*	5.8	2.3
Histone H2A type 1-C	*HIST1H2AC*	3.9	1.2
HLA class I histocompatibility antigen, A-2 alpha chain	*HLA-A*	detected in EV only	
Hyaluronan and proteoglycan link protein 1	*HAPLN1*	1.6	3.8
Hyaluronan and proteoglycan link protein 4	*HAPLN4*	detected in EV only	
Immunoglobulin superfamily containing leucine-rich repeat protein	*ISLR*	not detected in CM	
Interstitial collagenase	*MMP1*	0.4	2.4
Keratin, type II cytoskeletal 6B	*KRT6B*	0.7	11.2
Laminin subunit alpha-2	*LAMA2*	9.9	2.0
Laminin subunit alpha-4	*LAMA4*	2.1	2.3
Laminin subunit alpha-5	*LAMA5*	4.4	2.1
Laminin subunit beta-2	*LAMB2*	6.5	1.9

Laminin subunit gamma-1	*LAMC1*	2.2	1.2
Latent-transforming growth factor beta-binding protein 1	*LTBP1*	5.0	3.7
Latent-transforming growth factor beta-binding protein 2	*LTBP2*	9.4	1.2
Legumain	*LGMN*	2.1	1.0
Matrix-remodeling-associated protein 5	*MXRA5*	2.2	0.2
Metalloproteinase inhibitor 2	*TIMP2*	2.1	1.5
Myosin-9	*MYH9*	2.3	0.6
Myristoylated alanine-rich C-kinase substrate	*MARCKS*	10.3	11.2
N-acetylgalactosamine-6-sulfatase	*GALNS*	detected in EV only	
Nascent polypeptide-associated complex subunit alpha, muscle-specific form	*NACA*	not detected in CM	
Netrin-G1	*NTNG1*	detected in SF only	
Neudesin	*NENF*	0.7	2.4
Nidogen-1	*NID1*	3.3	0.9
Olfactomedin-like protein 3	*OLFML3*	2.3	1.4
Pappalysin-1	*PAPPA*	3.1	1.3
Pentraxin-related protein PTX3	*PTX3*	3.9	0.9
Periostin	*POSTN*	22.0	1.5
Peroxidasin homolog	*PXDN*	2.2	0.3
Peroxiredoxin-4	*PRDX4*	detected in EV only	
Pigment epithelium-derived factor	*SERPINF1*	1.0	2.2
Plasma protease C1 inhibitor	*SERPING1*	2.4	1.2
Platelet-activating factor acetylhydrolase IB subunit beta	*PAFAH1B2*	detected in EV only	
Polyubiquitin-C	*UBC*	1.2	2.0
Proteasome subunit alpha type-1	*PSMA1*	detected in EV only	
Proteasome subunit alpha type-3	*PSMA3*	7.4	2.1
Proteasome subunit alpha type-4	*PSMA4*	detected in SF only	
Proteasome subunit alpha type-6	*PSMA6*	1.5	2.0
Proteasome subunit beta type-4	*PSMB4*	3.5	1.0
Protein disulfide-isomerase	*P4HB*	4.5	2.5
Protein NOV homolog	*NOV*	detected in SF only	
Protein S100-A11	*S100A11*	0.9	3.4
Protein S100-A4	*S100A4*	0.7	2.3
Protein S100-A6	*S100A6*	1.9	3.4
Protein-lysine 6-oxidase	*LOX*	2.6	0.2
Ras-related protein Rab-6A	*RAB6A*	detected in EV only	
Retinoic acid receptor responder protein 1	*RARRES1*	4.3	1.9
Serglycin	*SRGN*	1.0	4.2
Serine protease 23	*PRSS23*	detected in SF only	
Soluble scavenger receptor cysteine-rich domain-containing protein SSC5D	*SSC5D*	3.4	1.3
SPARC	*SPARC*	3.0	1.6

Spectrin alpha chain, non-erythrocytic 1	*SPTAN1*	4.4	0.7
Spectrin beta chain, non-erythrocytic 1	*SPTBN1*	3.5	0.7
Stanniocalcin-2	*STC2*	detected in EV only	
Sushi repeat-containing protein SRPX2	*SRPX2*	2.5	1.1
Sushi, nidogen and EGF-like domain-containing protein 1	*SNED1*	3.5	1.3
Sushi, von Willebrand factor type A, EGF and pentraxin domain-containing protein 1	*SVEP1*	9.7	0.7
Syndecan-4	*SDC4*	2.7	0.8
T-complex protein 1 subunit eta	*CCT7*	detected in SF only	
T-complex protein 1 subunit zeta	*CCT6A*	2.3	1.5
Tenascin	*TNC*	11.9	2.2
Testican-1	*SPOCK1*	not detected in CM	
Thrombospondin-2	*THBS2*	3.3	1.0
Thrombospondin-3	*THBS3*	2.5	1.5
Thy-1 membrane glycoprotein	*THY1*	detected in EV only	
Transaldolase	*TALDO1*	4.0	6.7
Transitional endoplasmic reticulum ATPase	*VCP*	detected in EV only	
Translocon-associated protein subunit delta	*SSR4*	detected in EV only	
Triosephosphate isomerase	*TPI1*	1.2	3.8
Tubulin beta-4B chain	*TUBB4B*	detected in SF only	
Ubiquitin-like modifier-activating enzyme 1	*UBA1*	4.3	1.6
Vimentin	*VIM*	2.1	1.0
Xylosyltransferase 1	*XYLT1*	detected in EV only	
1,4-alpha-glucan-branching enzyme	*GBE1*	not identified in CM	
14-3-3 protein beta/alpha	*YWHAB*	2.2	1.8
14-3-3 protein eta	*YWHAH*	4.0	2.1
40S ribosomal protein S16	*RPS16*	detected in EV only	
40S ribosomal protein S3	*RPS3*	4.1	2.3
40S ribosomal protein S8	*RPS8*	2.8	2.3
6-phosphogluconate dehydrogenase, decarboxylating	*PGD*	detected in SF only	
60 kDa heat shock protein, mitochondrial	*HSPD1*	detected in SF only	
60 kDa heat shock protein, mitochondrial	*HSPD1*	detected in SF only	
60S ribosomal protein L14	*RPL14*	detected in EV only	
60S ribosomal protein L5	*RPL5*	3.0	1.6
78 kDa glucose-regulated protein	*HSPA5*	2.5	1.1
Actin-related protein 2/3 complex subunit 4	*ARPC4*	1.2	2.5
Adenylyl cyclase-associated protein 1	*CAP1*	detected in SF only	
Adipocyte enhancer-binding protein 1	*AEBP1*	3.5	0.4
Agrin	*AGRN*	2.0	1.8
Alpha-actinin-1	*ACTN1*	not detected in CM	
Alpha-actinin-4	*ACTN4*	5.3	2.8
Angiopoietin-related protein 2	*ANGPTL2*	3.5	1.0

Angiopoietin-related protein 4	*ANGPTL4*	2.8	1.4
Annexin A1;Annexin	*ANXA1*	4.0	1.4
Annexin A2;Annexin	*ANXA2*	5.2	1.6
Annexin A5;Annexin	*ANXA5*	2.1	0.2
Brain acid soluble protein 1	*BASP1*	4.5	3.7
Calpain-2 catalytic subunit	*CAPN2*	detected in EV only	
Calreticulin	*CALR*	4.3	3.8
Carbohydrate sulfotransferase 14	*CHST14*	not detected in CM	
Coiled-coil domain-containing protein 80	*CCDC80*	3.2	1.2
Collagen alpha-1(I) chain	*COL1A1*	2.6	2.0
Collagen alpha-1(III) chain	*COL3A1*	2.7	2.3
Collagen alpha-1(XII) chain	*COL12A1*	2.1	1.2
Collagen alpha-2(IV) chain	*COL4A2*	3.9	1.3
Collagen alpha-2(V) chain	*COL5A2*	8.6	1.7
Collagen alpha-2(VI) chain	*COL6A2*	3.0	1.5
Collectin-12	*COLEC12*	0.2	2.4
Complement component 1 Q subcomponent-binding protein, mitochondrial	*C1QBP*	0.8	4.8
Cytoplasmic dynein 1 heavy chain 1	*DYNC1H1*	detected in SF only	
Cytoskeleton-associated protein 4	*CKAP4*	detected in EV only	
Dickkopf-related protein 3	*DKK3*	1.5	2.1
Dihydropyrimidinase-related protein 3	*DPYSL3*	2.2	1.3
Ectonucleotide pyrophosphatase/phosphodiesterase family member 2	*ENPP2*	0.7	2.2
EGF-containing fibulin-like extracellular matrix protein 2	*EFEMP2*	11.6	1.2
EMILIN-1	*EMILIN1*	2.6	0.4
Endoplasmin	*HSP90B1*	2.7	1.1
Endosialin	*CD248*	1.9	3.3
Eukaryotic translation initiation factor 2 subunit 3	*EIF2S3*	detected in SF only	
Extracellular serine/threonine protein kinase FAM20C	*FAM20C*	4.3	0.7
Extracellular sulfatase Sulf-1	*SULF1*	2.5	0.4
F-actin-capping protein subunit alpha-1	*CAPZA1*	detected in SF only	
Fibulin-1	*FBLN1*	3.3	0.1
Fibulin-5	*FBLN5*	3.9	1.1
Filamin-A	*FLNA*	5.0	2.1
Fructose-bisphosphate aldolase C	*ALDOC*	not detected in CM	
Glucosamine-6-phosphate isomerase 1	*GNPDA1*	detected in EV only	
Glucose-6-phosphate 1-dehydrogenase	*G6PD*	detected in EV only	
Glutathione S-transferase omega-1	*GSTO1*	5.4	1.1
Golgi apparatus protein 1	*GLG1*	detected in EV only	
Growth arrest-specific protein 6	*GAS6*	detected in SF only	
Growth arrest-specific protein 6	*GAS6*	detected in SF only	

Heat shock cognate 71 kDa protein	*HSPA8*	4.6	1.0
Heat shock protein HSP 90-beta	*HSP90AB1*	3.4	3.3
Hepatocyte growth factor	*HGF*	detected in EV only	
Heterogeneous nuclear ribonucleoprotein A1	*HNRNPA1*	5.8	2.3
Histone H2A type 1-C	*HIST1H2AC*	3.9	1.2
HLA class I histocompatibility antigen, A-2 alpha chain	*HLA-A*	detected in EV only	
Hyaluronan and proteoglycan link protein 1	*HAPLN1*	1.6	3.8
Hyaluronan and proteoglycan link protein 4	*HAPLN4*	detected in EV only	
Immunoglobulin superfamily containing leucine-rich repeat protein	*ISLR*	not detected in CM	
Interstitial collagenase	*MMP1*	0.4	2.4
Keratin, type II cytoskeletal 6B	*KRT6B*	0.7	11.2
Laminin subunit alpha-2	*LAMA2*	9.9	2.0
Laminin subunit alpha-4	*LAMA4*	2.1	2.3
Laminin subunit alpha-5	*LAMA5*	4.4	2.1
Laminin subunit beta-2	*LAMB2*	6.5	1.9
Laminin subunit gamma-1	*LAMC1*	2.2	1.2
Latent-transforming growth factor beta-binding protein 1	*LTBP1*	5.0	3.7
Latent-transforming growth factor beta-binding protein 2	*LTBP2*	9.4	1.2
Legumain	*LGMN*	2.1	1.0
Matrix-remodeling-associated protein 5	*MXRA5*	2.2	0.2
Metalloproteinase inhibitor 2	*TIMP2*	2.1	1.5
Myosin-9	*MYH9*	2.3	0.6
Myristoylated alanine-rich C-kinase substrate	*MARCKS*	10.3	11.2
N-acetylgalactosamine-6-sulfatase	*GALNS*	detected in EV only	
Nascent polypeptide-associated complex subunit alpha, muscle-specific form	*NACA*	not detected in CM	
Netrin-G1	*NTNG1*	detected in SF only	
Neudesin	*NENF*	0.7	2.4
Nidogen-1	*NID1*	3.3	0.9
Olfactomedin-like protein 3	*OLFML3*	2.3	1.4
Pappalysin-1	*PAPPA*	3.1	1.3
Pentraxin-related protein PTX3	*PTX3*	3.9	0.9
Periostin	*POSTN*	22.0	1.5
Peroxidasin homolog	*PXDN*	2.2	0.3
Peroxiredoxin-4	*PRDX4*	detected in EV only	
Pigment epithelium-derived factor	*SERPINF1*	1.0	2.2
Plasma protease C1 inhibitor	*SERPING1*	2.4	1.2
Platelet-activating factor acetylhydrolase IB subunit beta	*PAFAH1B2*	detected in EV only	
Polyubiquitin-C	*UBC*	1.2	2.0
Proteasome subunit alpha type-1	*PSMA1*	detected in EV only	
Proteasome subunit alpha type-3	*PSMA3*	7.4	2.1

Proteasome subunit alpha type-4	*PSMA4*	detected in SF only	
Proteasome subunit alpha type-6	*PSMA6*	1.5	2.0
Proteasome subunit beta type-4	*PSMB4*	3.5	1.0
Protein disulfide-isomerase	*P4HB*	4.5	2.5
Protein NOV homolog	*NOV*	detected in SF only	
Protein S100-A11	*S100A11*	0.9	3.4
Protein S100-A4	*S100A4*	0.7	2.3
Protein S100-A6	*S100A6*	1.9	3.4
Protein-lysine 6-oxidase	*LOX*	2.6	0.2
Ras-related protein Rab-6A	*RAB6A*	detected in EV only	
Retinoic acid receptor responder protein 1	*RARRES1*	4.3	1.9
Serglycin	*SRGN*	1.0	4.2
Serine protease 23	*PRSS23*	detected in SF only	
Soluble scavenger receptor cysteine-rich domain-containing protein SSC5D	*SSC5D*	3.4	1.3
SPARC	*SPARC*	3.0	1.6
Spectrin alpha chain, non-erythrocytic 1	*SPTAN1*	4.4	0.7
Spectrin beta chain, non-erythrocytic 1	*SPTBN1*	3.5	0.7
Stanniocalcin-2	*STC2*	detected in EV only	
Sushi repeat-containing protein SRPX2	*SRPX2*	2.5	1.1
Sushi, nidogen and EGF-like domain-containing protein 1	*SNED1*	3.5	1.3
Sushi, von Willebrand factor type A, EGF and pentraxin domain-containing protein 1	*SVEP1*	9.7	0.7
Syndecan-4	*SDC4*	2.7	0.8
T-complex protein 1 subunit eta	*CCT7*	detected in SF only	
T-complex protein 1 subunit zeta	*CCT6A*	2.3	1.5
Tenascin	*TNC*	11.9	2.2
Testican-1	*SPOCK1*	not detected in CM	
Thrombospondin-2	*THBS2*	3.3	1.0
Thrombospondin-3	*THBS3*	2.5	1.5
Thy-1 membrane glycoprotein	*THY1*	detected in EV only	
Transaldolase	*TALDO1*	4.0	6.7
Transitional endoplasmic reticulum ATPase	*VCP*	detected in EV only	
Translocon-associated protein subunit delta	*SSR4*	detected in EV only	
Triosephosphate isomerase	*TPI1*	1.2	3.8
Tubulin beta-4B chain	*TUBB4B*	detected in SF only	
Ubiquitin-like modifier-activating enzyme 1	*UBA1*	4.3	1.6
Vimentin	*VIM*	2.1	1.0
Xylosyltransferase 1	*XYLT1*	detected in EV only	

Table S2. Enrichment of CM fraction compared to EV and SF subfractions in secreted proteins.

Protein name	Gene name	CM fraction enrichment, fold	
		relative to EV subfraction	relative to SF subfraction
40S ribosomal protein S4, X isoform	*RPS4X*	only detected in CM	
6-phosphogluconolactonase	*PGLS*	0.2	1.3
60S acidic ribosomal protein P0	*RPLP0*	0.4	0.6
60S acidic ribosomal protein P2	*RPLP2*	0.5	0.7
60S ribosomal protein L27	*RPL27*	0.5	not detected in SF
A disintegrin and metalloproteinase with thrombospondin motifs 5	*ADAMTS5*	0.4	0.7
Acid ceramidase	*ASAH1*	0.3	0.6
Actin-related protein 2/3 complex subunit 1B	*ARPC1B*	only detected in CM	
Actin-related protein 2/3 complex subunit 4	*ARPC4*	1.2	2.5
Acyl-CoA-binding protein	*DBI*	0.2	0.2
Aldo-keto reductase family 1 member C31	*AKR1C3*	0.2	0.4
Aldose reductase	*AKR1B1*	0.4	0.2
Alpha-N-acetylglucosaminidase	*NAGLU*	0.5	0.8
Aspartate aminotransferase, cytoplasmic	*GOT1*	0.4	0.4
Aspartate aminotransferase, mitochondrial	*GOT2*	0.3	0.6
Attractin	*ATRN*	0.5	not detected in SF
Basement membrane-specific heparan sulfate proteoglycan core protein	*HSPG2*	0.4	0.9
Beta-1,4-galactosyltransferase 1	*B4GALT1*	0.4	0.1
Beta-1,4-glucuronyltransferase 1	*B4GAT1*	0.4	0.7
Beta-2-microglobulin	*B2M*	0.2	0.7
Beta-hexosaminidase subunit beta	*HEXB*	0.4	0.8
Biotinidase	*BTD*	0.5	0.4
C-C motif chemokine 2	*CCL2*	0.2	0.5
Cadherin-13	*CDH13*	0.4	1.3
Cadherin-2	*CDH2*	0.2	0.4
Carboxypeptidase Q	*CPQ*	0.4	0.2
Cathepsin Z	*CTSZ*	only detected in CM	
CD44 antigen	*CD44*	0.4	0.3
Cellular retinoic acid-binding protein 2	*CRABP2*	0.1	0.2
Chitinase-3-like protein 1	*CHI3L1*	0.2	1.4
Clathrin heavy chain 1	*CLTC*	0.3	0.7
Coactosin-like protein	*COTL1*	0.5	1.4
Coagulation factor X	*F10*	0.2	0.3
Collectin-12	*COLEC12*	0.2	2.4
Complement component 1 Q subcomponent-binding protein, mitochondrial	*C1QBP*	0.8	4.8
Complement factor B	*CFB*	0.5	0.6

Complement factor D	*CFD*	0.2	1.2
Deoxyribonuclease-2-alpha	*DNASE2*	0.6	1.7
Destrin	*DSTN*	0.3	0.5
Di-N-acetylchitobiase	*CTBS*	0.3	0.5
Disintegrin and metalloproteinase domain-containing protein 10	*ADAM10*	0.3	0.2
DnaJ homolog subfamily C member 3	*DNAJC3*	0.4	1.0
Endoplasmic reticulum aminopeptidase 1	*ERAP1*	0.4	0.1
Endoplasmic reticulum resident protein 44	*ERP44*	0.2	0.5
Endothelial protein C receptor	*PROCR*	0.2	0.0
Epididymal secretory protein E1	*NPC2*	0.2	0.2
Epididymis-specific alpha-mannosidase	*MAN2B2*	0.3	0.4
Eukaryotic translation initiation factor 3 subunit B	*EIF3B*	only detected in CM	
Exostosin-2	*EXT2*	only detected in CM	
Ezrin	*EZR*	0.3	0.5
F-actin-capping protein subunit beta	*CAPZB*	0.4	0.5
Fatty acid synthase	*FASN*	not detected in EV	0.6
Fumarate hydratase, mitochondrial	*FH*	0.3	0.4
Gelsolin	*GSN*	0.9	1.8
Glucose-6-phosphate isomerase	*GPI*	0.5	0.3
Glutathione synthetase	*GSS*	0.6	1.4
GTP-binding nuclear protein Ran	*RAN*	0.5	not detected in SF
Heat shock 70 kDa protein 4	*HSPA4*	0.2	0.6
Heat shock protein HSP 90-alpha	*HSP90AA1*	0.5	not detected in SF
Hepatoma-derived growth factor	*HDGF*	0.2	1.5
Heterogeneous nuclear ribonucleoproteins A2/B1	*HNRNPA2B1*	0.5	0.9
High mobility group protein B1	*HMGB1*	0.5	not detected in SF
Histone H2A type 1-C	*HIST1H2AC*	3.9	1.2
Insulin-like growth factor-binding protein 3	*IGFBP3*	0.4	0.7
Insulin-like growth factor-binding protein 7	*IGFBP7*	0.4	0.4
Interleukin-6	*IL6*	0.2	0.4
Interstitial collagenase	*MMP1*	0.4	2.4
Isocitrate dehydrogenase [NADP] cytoplasmic	*IDH1*	0.2	0.3
Keratin, type II cytoskeletal 6B	*KRT6B*	0.7	11.2
Lactoylglutathione lyase	*GLO1*	0.2	0.4
Leukocyte elastase inhibitor	*SERPINB1*	0.2	0.2
Lysosomal Pro-X carboxypeptidase	*PRCP*	0.2	0.3
Lysosomal protective protein	*CTSA*	0.1	0.1
Macrophage colony-stimulating factor 1	*CSF1*	0.5	0.9
Macrophage migration inhibitory factor	*MIF*	0.1	0.1
Major prion protein	*PRNP*	0.2	0.1
Malate dehydrogenase, mitochondrial	*MDH2*	0.4	0.7

Matrix-remodeling-associated protein 8	*MXRA8*	0.4	0.4
Metalloproteinase inhibitor 1	*TIMP1*	0.4	0.3
Meteorin-like protein	*METRNL*	0.2	0.9
Microfibril-associated glycoprotein 4	*MFAP4*	0.3	0.1
Myosin regulatory light chain 12B	*MYL12B*	0.2	0.8
N-acetylglucosamine-1-phosphotransferase subunit gamma	*GNPTG*	0.1	0.1
N-acetylglucosamine-6-sulfatase	*GNS*	0.2	1.3
Neudesin	*NENF*	0.7	2.4
Nicotinamide phosphoribosyltransferase	*NAMPT*	0.5	0.6
Nuclease-sensitive element-binding protein 1	*YBX1*	2.6	not detected in SF
Nucleotide exchange factor SIL1	*SIL1*	0.2	0.3
Olfactomedin-like protein 1	*OLFML1*	0.5	1.2
Peptidase inhibitor 16	*PI16*	0.2	0.5
Peptidyl-glycine alpha-amidating monooxygenase	*PAM*	0.3	0.5
Peptidyl-prolyl cis-trans isomerase B	*PPIB*	0.4	0.4
Peptidyl-prolyl cis-trans isomerase C	*PPIC*	0.1	0.2
Peroxiredoxin-2	*PRDX2*	0.2	0.5
Peroxiredoxin-5, mitochondrial	*PRDX5*	0.2	0.4
Phosphoglycerate kinase 1	*PGK1*	0.3	0.2
Phosphoserine aminotransferase	*PSAT1*	0.4	0.6
Plasminogen activator inhibitor 2	*SERPINB2*	0.3	0.2
Polyubiquitin-C	*UBC*	1.2	2.0
Procollagen C-endopeptidase enhancer 1	*PCOLCE*	0.3	0.5
Prosaposin	*PSAP*	0.4	0.8
Prostaglandin reductase 1	*PTGR1*	0.5	not detected in SF
Proteasome activator complex subunit 1	*PSME1*	0.2	0.3
Proteasome subunit beta type-3	*PSMB3*	0.2	0.4
Protein FAM20A	*FAM20A*	0.1	0.2
Protein FAM3C	*FAM3C*	0.5	0.1
Protein S100-A10	*S100A10*	0.1	0.1
Protein S100-A11	*S100A11*	0.9	3.4
Protein S100-A13	*S100A13*	0.1	0.2
Protein S100-A4	*S100A4*	0.7	2.3
Purine nucleoside phosphorylase	*PNP*	0.2	0.4
Pyruvate kinase PKM	*PKM*	0.8	0.6
Rab GDP dissociation inhibitor beta	*GDI2*	0.5	1.1
Ras suppressor protein 1	*RSU1*	0.1	0.3
Receptor-type tyrosine-protein phosphatase gamma	*PTPRG*	0.2	0.6
Ribonuclease 4	*RNASE4*	only detected in CM	
Ribonuclease inhibitor	*RNH1*	only detected in CM	
Ribonuclease T2	*RNASET2*	0.4	not detected in SF
Selenium-binding protein 1	*SELENBP1*	0.3	0.7

Semaphorin-3D	*SEMA3D*	0.2	0.6
Semaphorin-7A	*SEMA7A*	0.2	1.6
Serpin B7	*SERPINB7*	0.2	0.4
SH3 domain-binding glutamic acid-rich-like protein	*SH3BGRL*	0.4	0.3
Sialate O-acetylesterase	*SIAE*	0.1	1.3
Sphingomyelin phosphodiesterase	*SMPD1*	0.4	0.4
Spondin-2	*SPON2*	only detected in CM	
Stathmin	*STMN1*	0.3	1.2
Stress-70 protein, mitochondrial	*HSPA9*	only detected in CM	
Stromal cell-derived factor 1	*CXCL12*	0.2	0.3
Superoxide dismutase [Cu-Zn]	*SOD1*	0.4	0.1
Superoxide dismutase [Mn], mitochondrial	*SOD2*	0.5	not detected in SF
Sushi repeat-containing protein SRPX	*SRPX*	1.5	not detected in SF
Syntenin-1	*SDCBP*	0.3	0.7
T-complex protein 1 subunit alpha	*TCP1*	not detected in SF	0.3
T-complex protein 1 subunit gamma	*CCT3*	0.4	0.6
Tetranectin	*CLEC3B*	0.4	1.1
Thioredoxin	*TXN*	0.4	0.5
Thioredoxin domain-containing protein 17	*TXNDC17*	0.1	0.2
Thioredoxin reductase 1, cytoplasmic	*TXNRD1*	0.5	0.1
Tissue factor pathway inhibitor	*TFPI*	0.4	0.9
Transcobalamin-2	*TCN2*	0.3	0.3
Transforming growth factor beta receptor type 3	*TGFBR3*	0.2	0.2
Transgelin-2	*TAGLN2*	0.5	not detected in SF
Translationally-controlled tumor protein	*TPT1*	0.3	1.2
Tripeptidyl-peptidase 1	*TPP1*	0.4	0.2
Tryptophan-tRNA ligase, cytoplasmic	*WARS*	0.4	1.0
Tumor necrosis factor receptor superfamily member 11B	*TNFRSF11B*	0.5	not detected in SF
Twisted gastrulation protein homolog 1	*TWSG1*	only detected in CM	
Ubiquitin thioesterase OTUB1	*OTUB1*	0.4	0.9
Ubiquitin-conjugating enzyme E2 N	*UBE2N*	0.4	0.6
UMP-CMP kinase	*CMPK1*	0.2	0.4
Urokinase-type plasminogen activator	*PLAU*	0.3	0.3
Vacuolar protein sorting-associated protein 35	*VPS35*	0.4	0.6
Vesicular integral-membrane protein VIP36	*LMAN2*	0.5	0.4
Vitamin K-dependent protein S	*PROS1*	0.5	0.7

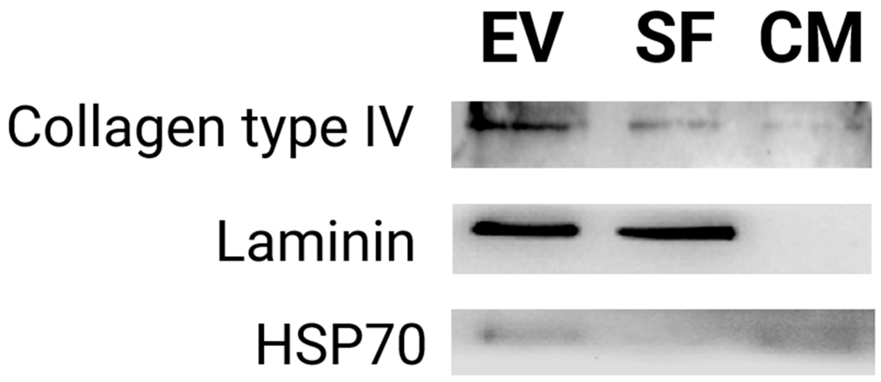

Figure S3. Verification of MCS secretome fractions proteomic analysis results by Western blotting. Immunochemical staining for collagen type IV (EV/CM fold=3.9, SF/CM fold=1.3), laminin (EV/CM fold>2.1, SF/CM fold>1.2) and HSP70 (EV/CM fold=0.9, SF/CM fold=0.4).

Table S3. Enrichment of CM fraction compared to EV and SF subfractions in secreted proteins regulating immune system signaling pathways.

**Protein name**	**Gene name**	**Netrophil degranulation** (REAC:R-HSA-6798695)	**IL-12 signaling** (REAC:R-HSA-9020591); **Gene and protein expression by JAK-STAT signaling after IL-12 stimulation** (REAC:R-HSA-8950505)	**Immune System** (REAC:R-HSA-168256)
Actin-related protein 2/3 complex subunit 1B	*ARPC1B*			+
Beta-1,4-galactosyltransferase 1	*B4GALT1*	+		+
Cadherin-2	*CDH2*			
Cathepsin Z	*CTSZ*	+		+
CD44 antigen	*CD44*	+		+
Disintegrin and metalloproteinase domain-containing protein 10	*ADAM10*	+		+
Endoplasmic reticulum aminopeptidase 1	*ERAP1*			+
Epididymal secretory protein E1	*NPC2*	+		+
F-actin-capping protein subunit beta	*CAPZB*			+
Glucose-6-phosphate isomerase	*GPI*	+		+
High mobility group protein B1	*HMGB1*	+		+
Insulin-like growth factor-binding protein 7	*IGFBP7*			
Interleukin-6	*IL6*			+
Isocitrate dehydrogenase [NADP] cytoplasmic	*IDH1*	+		+
Leukocyte elastase inhibitor	*SERPINB1*	+		+
Lysosomal Pro-X carboxypeptidase	*PRCP*	+		+
Lysosomal protective protein	*CTSA*	+		+

Macrophage migration inhibitory factor	*MIF*	+	+	+
Matrix-remodeling-associated protein 8	*MXRA8*			
Metalloproteinase inhibitor 1	*TIMP1*			+
Plasminogen activator inhibitor 2	*SERPINB2*		+	+
Proteasome activator complex subunit 1	*PSME1*			+
Proteasome subunit beta type-3	*PSMB3*			+
Protein disulfide-isomerase A3	*PDIA3*			+
Protein FAM20A	*FAM20A*			
Purine nucleoside phosphorylase	*PNP*	+		+
Ribonuclease T2	*RNASET2*	+		+
Stress-70 protein, mitochondrial	*HSPA9*		+	+
Superoxide dismutase [Cu-Zn]	*SOD1*		+	+
Superoxide dismutase [Mn], mitochondrial	*SOD2*		+	+
T-complex protein 1 subunit alpha	*TCP1*		+	+
Tumor necrosis factor receptor superfamily member 11B	*TNFRSF11 B*			+
Urokinase-type plasminogen activator	*PLAU*	+		+

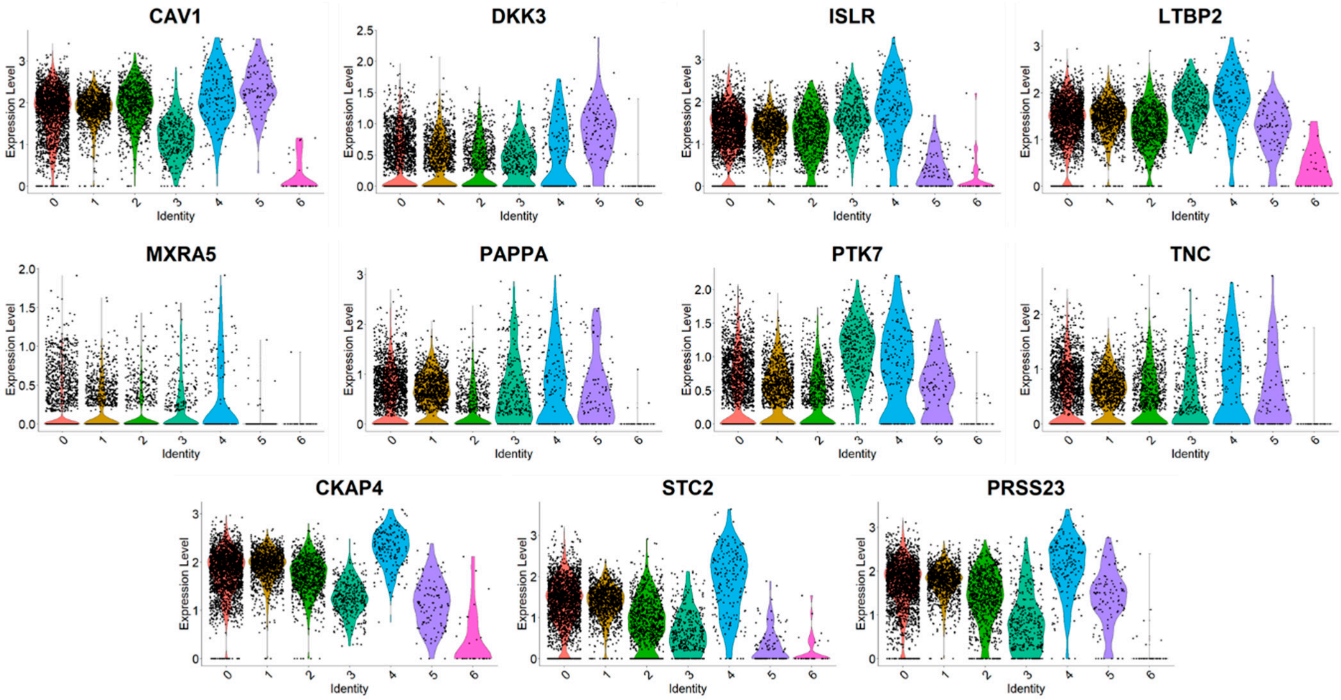

Figure S4. Expression of eleven potential antifibrotic genes in each cluster. Results of scRNAsec of adipose tissue derived MSCs.

Table S4. Number and proportion of cells of each cluster in Control sample and "Profibrotic conditions" sample. Results of scRNAsec of adipose tissue derived MSCs.

Cluster	Percent of cells in cluster of Control sample	Percent of cells in cluster of "Profibrotic conditions" sample	Number of cells in cluster	Number of cells of Control sample in cluster	Number of cells of "Profibrotic conditions" sample in cluster
0	48.2	36.81	2481	2139	342
1	28.23	3.66	1287	1253	34
2	18.77	7.21	900	833	67
3	0.14	41.01	387	6	381
4	3.85	0.86	179	171	8
5	0.59	8.29	103	26	77
6	0.23	2.15	30	10	20
